# The Noncoding RNA Expression Profile and the Effect of lncRNA AK126698 on Cisplatin Resistance in Non-Small-Cell Lung Cancer Cell

**DOI:** 10.1371/journal.pone.0065309

**Published:** 2013-05-31

**Authors:** Yong Yang, Hui Li, Shengcai Hou, Bin Hu, Jie Liu, Jun Wang

**Affiliations:** 1 Beijing Key Laboratory of Respiratory and Pulmonary Circulation, Capital Medical University, Beijing, China; 2 Department of Thoracic Surgery, Beijing Chao-Yang Hospital, Capital Medical University, Beijing, China; 3 Department of Physiology, Capital Medical University, Beijing, China; University of Barcelona, Spain

## Abstract

**Background:**

The efficacy of cisplatin-based chemotherapy in non-small-cell lung cancer is limited by the acquired drug resistance. Identification the RNAs related to the cisplatin resistance may help to improve clinical response rates.

**Methods:**

Microarray expression profiling of mRNAs, lncRNA and miRNA was undertaken in A549 cells and cisplatin resistant A549/CDDP cells. Differentially expressed mRNAs, lncRNAs and miRNAs, verified by realtime RT-PCR, were subjected to pathway analysis. Expression of NKD2 and β-catenin was assessed by realtime RT-PCR and western blot analysis. The effect of lncRNA AK126698 on cisplatin induced apoptosis was investigated by annexin-V/PI flow cytometry.

**Results:**

In total, 1471 mRNAs, 1380 lncRNAs and 25 miRNAs differentially expressed in A549/CDDP and A549 cells. Among them, 8 mRNAs, 8 lncRNAs and 5 miRNAs differentially expressed in gene chip analysis were validated. High-enrichment pathway analysis identified that some classical pathways participated in proliferation, differentiation, avoidance of apoptosis, and drug metabolism were differently expressed in these cells lines. Gene co-expression network identified many genes like FN1, CTSB, EGFR, and NKD2; lncRNAs including BX648420, ENST00000366408, and AK126698; and miRNAs such as miR-26a and let-7i potentially played a key role in cisplatin resistance. Among which, the canonical Wnt pathway was investigated because it was demonstrated to be targeted by both lncRNAs and miRNAs including lncRNA AK126698. Knockdown lncRNA AK126698 not only greatly decreased NKD2 which can negatively regulate Wnt/β-catenin signaling but also increased the accumulation and nuclear translocation of β-catenin, and significantly depressed apoptosis rate induced by cisplatin in A549 cells.

**Conclusion:**

Cisplatin resistance in non-small-cell lung cancer cells may relate to the changes in noncoding RNAs. Among these, AK126698 appears to confer cisplatin resistance by targeting the Wnt pathway.

## Introduction

Lung cancer is one of the most common human cancers worldwide and continues to be associated with the highest incidence and mortality rates of all cancers [1], [2]. According to the WHO GLOBOCAN project, 1.6 million new cases of lung cancer, accounting for 12.7% of the world’s total cancer incidence, were diagnosed in 2008 [3]. Non-small-cell lung cancer (NSCLC) accounts for approximately 85% of all lung cancer cases [4]. The most effective therapy for NSCLC is complete lung resection. However, the survival rate after complete lung resection is far from satisfactory and most patients are offered chemotherapy as an alternative, in particular cisplatin (CDDP; cis-diamminedichloroplatinum II)-based chemotherapy.

Cisplatin primarily acts by causing DNA damage [5]. However, the ability of cancer cells to become resistant to CDDP remains a significant impediment to successful chemotherapy. Previous studies have proposed a number of potential mechanisms of cisplatin resistance [6]. But, there is an ongoing need to pinpoint the exact mechanisms involved in order to find new targets to prevent drug resistance.

The rapid development of molecular biology makes it possible to detect molecular differences between different cells. This approach may provide important clues concerning the drug resistance. Understanding the relationships between cisplatin resistance and molecular changes will help to predict the cisplatin resistance in advance and to improve the efficacy of therapeutic intervention.

The human transcriptome comprises large numbers of protein-coding messenger RNAs (mRNAs), together with a large set of nonprotein coding transcripts including long noncoding RNAs and microRNA that have structural, regulatory, or unknown functions [7], [8]. Long noncoding RNAs (lncRNAs) which are characterized by the complexity and diversity of their sequences and mechanisms of action are distinct from small RNAs or structural RNAs and are thought to function as either primary or spliced transcripts [9]. Altered lncRNA levels have been shown to result in aberrant expression of gene products that may contribute to different disease states including cancer [10], [11]. However, the overall pathophysiological contribution of lncRNAs to cisplatin resistance remains largely unknown.

MicroRNAs (miRNAs) are a family of ∼22nt small, non-coding, endogenous, single-stranded RNAs that regulate gene expression. Mature miRNAs and Argonaute (Ago) proteins form the RNA-induced silencing complex (RISC), which mediates post-transcriptional gene silencing through induction of mRNA degradation or translational inhibition [12]. Some miRNAs had been found play important role in cisplatin resistance [13], [14], but more research is needed to explore the relationships between miRNAs, lncRNAs and mRNAs in the cancer biology process.

The Wnt/β-catenin canonical signaling pathway was previously regarded as playing a central roll in determining cell fate [15]. The Wnt pathway has now been found to be altered in many types of cancer [16]. Following binding of Wnt to its receptor, Dishevelled proteins (Dsh/Dvl) become activated, leading to the inactivation of the axin/adenomatous polyposis coli (APC)/glycogen synthase kinase (GSK)3β complex that prevents the degradation of β-catenin [17]. This results in stabilized β-catenin being translocated to the nucleus where it binds to members of the T cell factor/lymphoid enhancer-binding factor (TCF/LEF) family of transcriptional factors, and is able to modulate the expression of a broad range of target genes to regulate cell fates.

Wnt-β-catenin pathway [18] are precisely controlled by a number of regulators. Among them, the naked cuticle (NKD) family includes Drosophila naked cuticle and its two vertebrate orthologs NKD1 and NKD2 have been shown to negatively regulate canonical Wnt signaling by binding to Dvl. However, whether the Wnt pathway is involved in cisplatin resistance or its regulation is still unknown.

In this study, lncRNA, miRNA and mRNA expression profiles were compared in wild type A549 and cisplatin resistance cell lines A549/CDDP using Gene Chip technology. An integrative analysis combining changes in the three groups of RNA within different genetic networks was used to identify genes and pathways that may be related to cisplatin resistance in NSCLC. Experiments with lncRNA AK126698 knockdown were used to observe its impact on the canonical Wnt pathway and cell responses to cisplatin.

## Materials and Methods

### Cell culture

Human lung adenocarcinoma cell line A549 and cisplatin-resistant variant cell line A549/CDDP were purchased from the Peking Union Medical College, Beijing, China. A549 and A549/CDDP cells were maintained in RPMI-1640 medium (life technologies) supplemented with 10% fetal calf serum (Gibco, Gran Island, NY, USA) in a humid atmosphere containing 5% CO_2_ at 37°C. The A549/CDDP cell medium additionally contained 2 mg/L cisplatin in order to maintain its drug-resistant phenotype. Cells in the logarithmic phase of growth were used for all experiments.

### LncRNA microarray

Briefly, A549 and A549/CDDP cells were used to synthesize double-stranded complementary DNA (cDNA). Double-stranded cDNA was labeled and hybridized to the 8×60 K LncRNA Expression Microarray (Arraystar, Rockville, MD). The lncRNA expression microarray used in this study mainly classifies its probes as the following subtype: 1). Enhancer LncRNAs: Contains profiling data of all LncRNAs with enhancer-like function [19]. 2). Rinn lincRNAs: Contains profiling data of all lincRNAs based on John Rinn's papers [20], [21]. 3). HOX cluster: Contains profiling data of all probes in the four HOX loci, targeting 407 discrete transcribed regions, lncRNAs and coding transcripts [22]. 4). LincRNAs nearby coding gene: Contains the differentially expressed lincRNAs and nearby coding gene pairs (distance <300 kb). 5). Enhancer LncRNAs nearby coding gene: Contains the differentially expressed enhancer-like LncRNAs and their nearby coding genes (distance <300 kb). After hybridization and washing, processed slides were scanned with the Agilent DNA Microarray Scanner (part number G2505B). Agilent Feature Extraction software (version 10.7.3.1) was used to analyze acquired array images. Quantile normalization and subsequent data processing were performed with using the GeneSpring GX v11.5.1 software package (Agilent Technologies). Each cell line performed lncRNA microarray in triplicates.

### MiRNA microarray

Microarray profiling for miRNA was performed using Affymetrix GeneChip miRNA arrays (Santa Clara, CA, USA) according to manufacturer's recommended protocol. Briefly, 1 µg of total RNA from the cells was labeled by polyA polymerase using the Genisphere FlashTag HSR kit following the manufacturer's recommendations (Genisphere, Hatfield, PA). RNA was hybridized to the Affymetrix miRNA array as recommended by the vendor. Standard Affymetrix array cassette staining, washing and scanning was performed using the post-hybridization kit (#900720; Affymetrix) and GeneChip Scanner 3000. Feature extraction was performed using Affymetrix Command Console software. The raw data were processed in the following sequence: background detection was followed by RMA global background correlation, quantile normalization, median estimation and log2-transformation using the miRNA QC software tool (Affymetrix). Each cell line performed miRNA microarray in triplicates.

### In vitro drug sensitivity assay

Cells were seeded in 96-well plates at a density of 5×10^3^ cells/well and incubated overnight at 37°C. The cells were incubated with different concentrations of cisplatin for 48 h at 37°C. After addition of 20 µL of CellTiter 96R AQueous One solution (Promega) to each well, plates were incubated for 2.5 h at 37°C. Absorbance of each well at 490 nm (A490) was read using a spectrophotometer. The concentration at which each drug produced 50% inhibition of growth (IC50) was estimated from relative survival curves. Three independent experiments were performed in six duplicate wells.

### Realtime RT-PCR

Real-time RT-PCR was used to verify differential expression of 16 genes that were detected by the LncRNA Expression Microarray. The cDNA was synthesized using reverse transcriptase (TaKaRa), oligo (dT) primers with 1 µg RNA from the same samples as those used in the microarray. The primers used are listed in Table S1. Each real-time RT-PCR reaction (in 20 µL) contained 2.5×SYBR Green Realtime PCR Master Mix (TIANGEN), 0.5 µM primers and 0.5 µL of template cDNA. The cycling conditions consisted of an initial, single cycle of 2 min at 94°C, followed by 40 cycles of 15 s at 94°C, 20 s at 63°C, and 30 s at 68°C. PCR amplifications were performed in three duplicates for each sample. Gene expression levels were quantified relative to the expression of 18S using an optimized comparative Ct (ΔΔCt) value method. The differences in gene expression levels between groups were compared using the Student's t-test. P-values <0.05 were considered statistically significant.

### QRT-PCR of miRNA

Bulge-loop™ miRNA qRT-PCR Primer Sets (one RT primer and a pair of qPCR primers for each set) specific for miR-17, miR-21, miR-138, miR-194, and miR-let-7i were designed by RiboBio (Guangzhou, China). Briefly, the total RNA was extracted using a MiRNeasy Mini Kit (QIAGEN). The miRNA bulge-loop was reverse transcribed with the Quantscript RT Kit (TIANGEN). Each real-time RT-PCR reaction (in 25 µL) contained 2×SuperReal PreMix (TIANGEN), 10 µM primers, and 1 µL of template cDNA. The cycling conditions consisted of an initial, single cycle of 3 min at 95°C, followed by 40 cycles of 10 s at 95°C, 20 s at 60°C and 30 s at 70°C. PCR amplifications were performed in three duplicates for each sample. The relative amount of miRNAs was normalized against U6 snRNA, and the fold change for each miRNA was calculated by the 2^−ΔΔCt^ method. P-values <0.05 were considered statistically significant.

### Small interfering RNA (siRNA)

To estimate inhibition of AK126698, 50 nM of AK126698 siRNA (Shanghai Genepharma, China) were transfected into A549 cells using Lipofectamine 2000 reagent according to the manufacturer’s instructions. Cells transfected with the transfection agent and scramble-control siRNA (negative control) were used as controls. The cells were harvested 48 hours after transfection. Four pairs of siRNA were named siRNA AK126698-291, siRNA AK126698-341, siRNA AK126698-424 and siRNA AK126698-1492, respectively. Compared with control, only siRNA siRNA AK126698-291 and AK126698-424 successfully decrease the expression level of lncRNA AK126698. The sequences of AK126698 siRNA and scramble control siRNA are listed in Table S2.

### Western blot analysis

A549 cells were plated in 6-well plates (2×10^5^ cells/well). 48 hours after transfection of AK126698 siRNA or negative control, the cells were harvested and homogenized with lysis buffer. Total protein was separated by denaturing 10% SDS–polyacrylamide gel electrophoresis. Detection was performed with Odyssey system (Gene Company Limited, USA). The primary antibodies for β-catenin, phospho-β-catenin (Ser675) and β-actin were purchased from Santa Cruz Biotechnology, Cell Signaling Technology and Sigma-Aldrich, respectively. The primary antibodies for Cell cycle pathway phospho-cdc2 (Tyr15), phospho-Rb (ser807), phospho-Chk2 (Thr68) and phospho-p53 (ser15) and for the MAPK signaling pathway phospho-SAPK/JNK (Thr183/Tyr185) and phospho-p44/42 MAPK (Erk1/2) (Thr202/Tyr204) were purchased from Cell signaling Technology. Antibody dilutions were 1∶2000 for β-catenin, 1∶1000 for phospho-β-catenin, 1∶1000 for phospho-Rb (ser807), 1∶1000 for phospho-Chk2 (Thr68), 1∶1000 for phospho-cdc2 (Tyr15), 1∶200 for phospho-p53 (ser15), 1∶200 for phospho-SAPK/JNK (Thr183/Tyr185), 1∶200 for phospho-p44/42 MAPK (Erk1/2) (Thr202/Tyr204) and 1∶5000 for β-actin. Protein levels were normalized to β-actin and changes were determined.

### Flow Cytometry

Cells were plated in 6-well plates (2×10^5^ cells/well). 24 hours after the transfection of siRNA AK126698 as described above, A549 cells were treated by CDDP at a final concentration of 10 mg/L. 24 hours after the treatment of CDDP, flow cytometry was used to detect apoptosis of the transfected A549 cells by determining the relative amount of Annexin V-FITC-positive-PI-negative cells.

### Data Analysis

Each experiment was repeated at least three times. Numerical data were presented as means and standard errors (± SEM). Differences between means were analyzed using Student’s t test. All statistical analyses were performed using SPSS11.0 software (Chicago, IL).

#### Significant Differential Gene Analysis

The random variance model (RVM) t-test was used to identify differentially expressed genes for the control and experiment group. This model has more power than standard tests to pick up large changes in expression, without increasing the rate of false positives [23]. After the significant analysis and false discovery rate (FDR) analysis, we selected the differentially expressed genes according to predefined P-value thresholds (<0.05) [23], [24], [25]. The results of differentially expressed genes were subjected to unsupervised hierarchical clustering (Cluster 3.0) and TreeView analysis (Stanford University, Stanford, CA, USA).

#### MicroRNA targets prediction

Targets mRNAs of miRNAs were predicted based on TargetScan (http://www.targetscan.org/) version 5.2. TargetScan predicts biological targets of miRNAs by searching for the presence of conserved 8mer and 7mer sites that match the seed region of each miRNA [26]. Also identified are sites with mismatches in the seed region that are compensated by conserved 3' pairing [27]. In mammals, predictions are ranked based on the predicted efficacy of targeting as calculated using the context + scores of the sites alignments [28], [29]. TargetScanHuman considers matches to annotate human UTRs and their orthologs, as defined by UCSC whole-genome alignments. Conserved targeting has also been detected within open reading frames (ORFs).

#### Pathway analysis

Pathway analysis was used to identify significant pathways for the differential genes according to the Kyoto Encyclopedia of Genes and Genomes, Biocarta and Reatome databases. We also used Fisher’s exact and chi-square tests to select significant pathways. The threshold of significance was defined by P-value and FDR. The enrichment Re was given by: 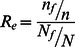
(R_e_ = ENRICHMENT), where 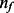 is the number of differential genes within the particular category, 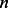 is the total number of genes within the same category, 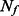 is the number of differential genes in the entire microarray, and 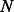 is the total number of genes in the microarray [30], [31], [32].

#### GeneRelNet (Coexpression network)

Gene coexpression networks were used to identify gene interactions [33]. Gene coexpression networks were built according to the normalized signal intensity of specific expression genes. For each pair of genes, we calculated the Pearson correlation coefficient and choose significant correlation pairs to construct the network [34].

Within the network analysis, the degree of gene centrality defined as the number of links from one node to another, was used to determine its relative importance [35]. K-cores were used for graph topology analysis. The k-core of a network was a subnetwork in which all nodes were connected to at least 'k' other genes in the subnetwork. Within a protein-protein interaction, k-core networks usually contain cohesive groups of proteins [35], [36].

## Results

### Microarray of A549 and A549/CDDP cell lines

**Figure 1:**
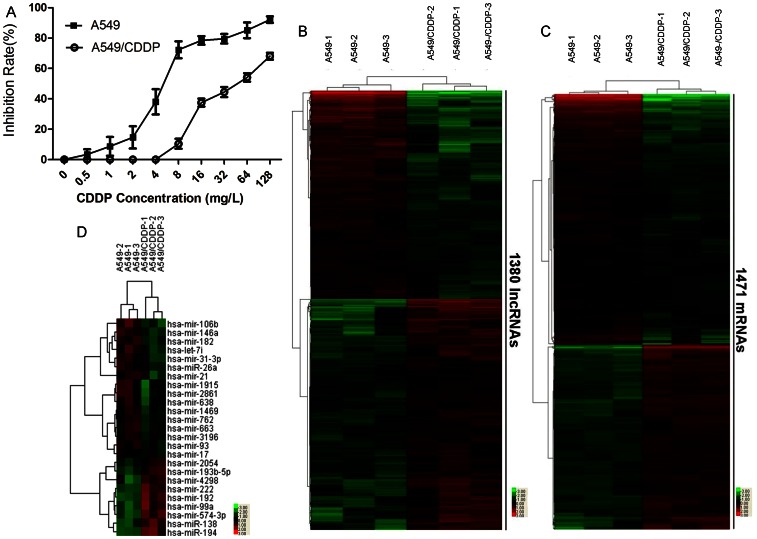
The RNA expression profiles and ciapatin resistance of A549 and A549/CDDP cells. Cells were treated with increasing concentrations of cisplatin (0.5 mg/L to 128 mg/L). 48 hours later, cell viability was measured by MTS (A). Heat maps show lncRNA (B), mRNA (C) and miRNAs (D) profiles that differentiate A549/CDDP from A549. Each sample was assayed in triplicate. Both down-regulated (green) and up-regulated (red) RNAs were identified in A549.

First, the cisplatin-resistance of A549/CDDP cell line was identified by evaluating the IC50-value of A549/CDDP against wild A549 cell line. As showed in Figure 1A, the IC50 of cisplatin for the drug-resistant A549/CDDP cell line was 17.06±0.68 mg/L which was 3.9 times higher than that of the wild type A549 cell line (4.36±0.78 mg/L). This result demonstrated that the A549/CDDP cells were more resistant to cisplatin than the wild type cells.

Then a gene chip study was performed in these two cell lines to investigate the possible RNA expression changes in cisplatin resistance in lung adenocarcinoma cells using the Arraystar probe dataset which included 33,045 lncRNAs and 30,215 coding transcripts. Hierarchical clustering showed systematic variations in the expression of lncRNAs and protein-coding RNAs between the two cell lines (Figure 1B and 1C). Compared with A549 cell line, 725 probes of lncRNAs increased and 655 probes decreased in the A549/CDDP cell line. In addition, 625 mRNA differential probe increases and 846 mRNA probe decreases were found in the A549/CDDP cell line.

To investigate if the microRNA expression was changed in cisplatin resistant cells, miRNA expression profiles were assessed using the Affymetrix miRNA gene chip which contained 1,105 pre-miRNAs and 1,105 mature-miRNAs probes. For further analysis we focused on mature-miRNAs. The results showed that in A549/CDDP cells, 16 miRNAs downregulated and 9 miRNAs upregulated in comparison with the A549 cell line (P<0.05; Figure 1D). The microarray data discussed in this article have been deposited in National Center for Biotechnology Information (NCBI) Gene Expression Omnibus (GEO) and are accessible through (GEO) Series accession number GSE43494 (http://www.ncbi.nlm.nih.gov/geo/query/acc.cgi?acc=GSE43494).

### Validation of the microarray data using qPCR

**Figure 2:**
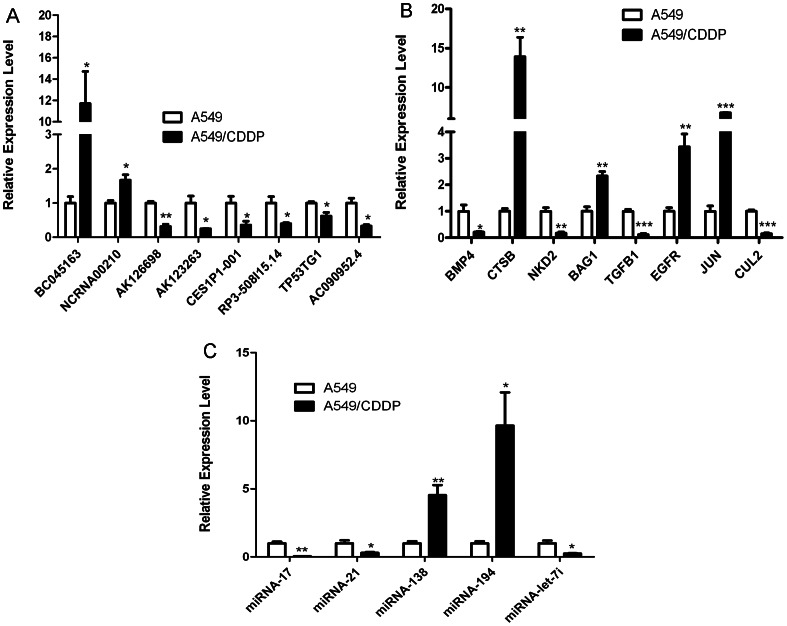
Validation of microarray data by realtime RT-PCR. The relative amount of each mRNA (A) and lncRNA (B) was normalized to 18S rRNA, and each miRNA (C) was normalized to U6 snRNA. Data in histograms are means±SD, *P<0.05, **P<0.01, ***P<0.001 compared with A549 (t test).

To validate the microarray analysis findings, the expression level of 8 mRNAs which are thought to play an important role in drug resistance and 8 lncRNAs which had correlations with antecedent mRNAs among the differential expression RNAs, were analyzed using quantitative real-time polymerase chain reaction (qRT-PCR). For lncRNA, the results showed that AK123263, CES1P1-001, RP3-508I15.14, AK126698, TP53TG1, and AC090952.4.1 decreased, whereas uc003bgl.1 and NCRNA00210 increased in A549/CDDP (all P <0.05; Figure 2A). For mRNA, the expression of BMP4, CTSB, NKD2, BAG1, TGFB1, EGFR, JUN and CUL2 showed statistically significant differences between the two cell lines (P <0.05; Figure 2B). All the qRT-PCR results above were consistent with the microarray. These results indicate that a set of lncRNAs and mRNAs are aberrantly expressed in cisplatin resistance cell lines.

5 miRNAs among those filtered were validated to be significantly different between the cisplatin resistant cell line A549/CDDP and the parental A549 cell line (P <0.05). As shown in (Figure 2C), the levels of miR-17, miR-21, and miR-let-7i were down-regulated in A549 rather than A549/CDDP, while the miR-138 and miR-194 expression was up-regulated. This finding is in agreement with the results of microarray hybridization.

### Microarray-based pathway analysis

**Figure 3:**
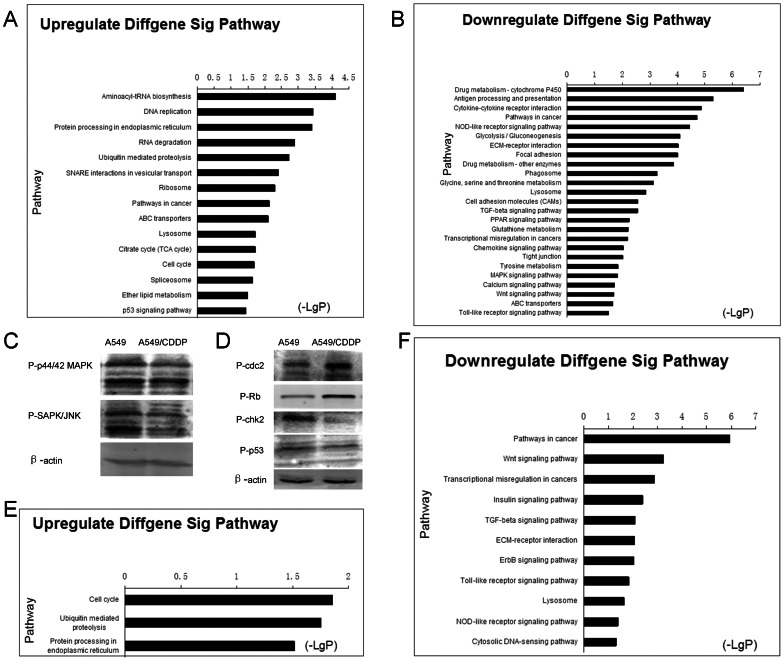
Signaling pathways of differentially expressed RNAs. Signaling pathways of differentially expressed upregulated mRNAs (A) and downregulated mRNAs (B). Western blot analysis of MAPK pathway (C) and Cell cycle pathway (D) levels in A549 cells and A549/CDDP cells. P-p44/42 MAPK, phosphor-p44/42 MAPK; P-SAPK/JNK, phosphor-SAPK/JNK; P-cdc2, phosphor-cdc2; P-Rb, phosphor-Rb; P-chk2, phosphor-chk2; P-p53, phosphor-p53. Signaling pathways on the intersection between differently expressed mRNAs and predicted target genes from differently expressed upregulated mRNAs (E) and downregulated mRNAs (F). Pathway analysis was predominantly based on the KEGG database. P-values <0.05 using the two-sided Fisher’s exact test were classed as being statistically significant. The vertical axis represents the pathway category and the horizontal axis represents the -log10 (p value) of these significant pathways.

Significant pathways of differential genes were compared with the KEGG database to further specify and identify target mRNAs among the 1,471 identified genes. These genes are shown in Figure 3A and 3B. The high-enrichment pathways targeted by overexpressed mRNAs were involved in aminoacyl-tRNA biosynthesis, DNA replication and protein processing in endoplasmic reticulum. In contrast, significant pathways corresponding to underexpressed mRNAs appeared to be responsible for drug metabolism, antigen processing and presentation and cytokine-cytokine receptor interactions. Among these, the maximum-enriched-pathways relating to proliferation and drug metabolism suggested a role in cisplatin resistance.

Cell cycle and MAPK signaling pathway were chose to verify the accuracy of the pathway analysis results. Compared with A549 cells, the key factors in Cell cycle phospho-cdc2 (Tyr15) and phospho-Rb (ser807/811) increased in protein level in A549/CDDP cells represent the upregulation of Cell cycle pathway. The decreased of cdc2 negative regulator phospho-Chk2 (Thr68) and phospho-p53 (ser15) reinforced this result. Also, the phospho-SAPK/JNK (Thr183/Tyr185) and phospho-p44/42 MAPK (Erk1/2) (Thr202/Tyr204) which are the main factors of MAPK signaling pathway, decreased in A549/CDDP cell line. And these results listed in Figure 3C and 3D.

The target mRNAs for differentially expressed miRNAs were predicted using TargetScan (http://www.targetscan.org/) and 7,814 relationships between them were observed (Table S3). The intersection set for the predicted target mRNAs and differentially expressed mRNAs mentioned above was selected. 497 relationships left after this step as shown in Table S4. Significant pathways for these genes (P <0.05) thought to be regulated by miRNAs according to the KEGG database were analyzed (Figure 3E and 3F). The significant pathways included Wnt signaling pathway, transcriptional misregulation in cancer and, insulin signaling pathways.

We screened out 21 differentially expressed mRNAs in cisplatin resistance cells (Table S5) that were negatively correlated and possibly regulated by miRNAs. These mRNAs were involved in 11 of the pathways that were thought to play important role in the cisplatin resistance.

### Establishment of gene co-expression network

**Figure 4:**
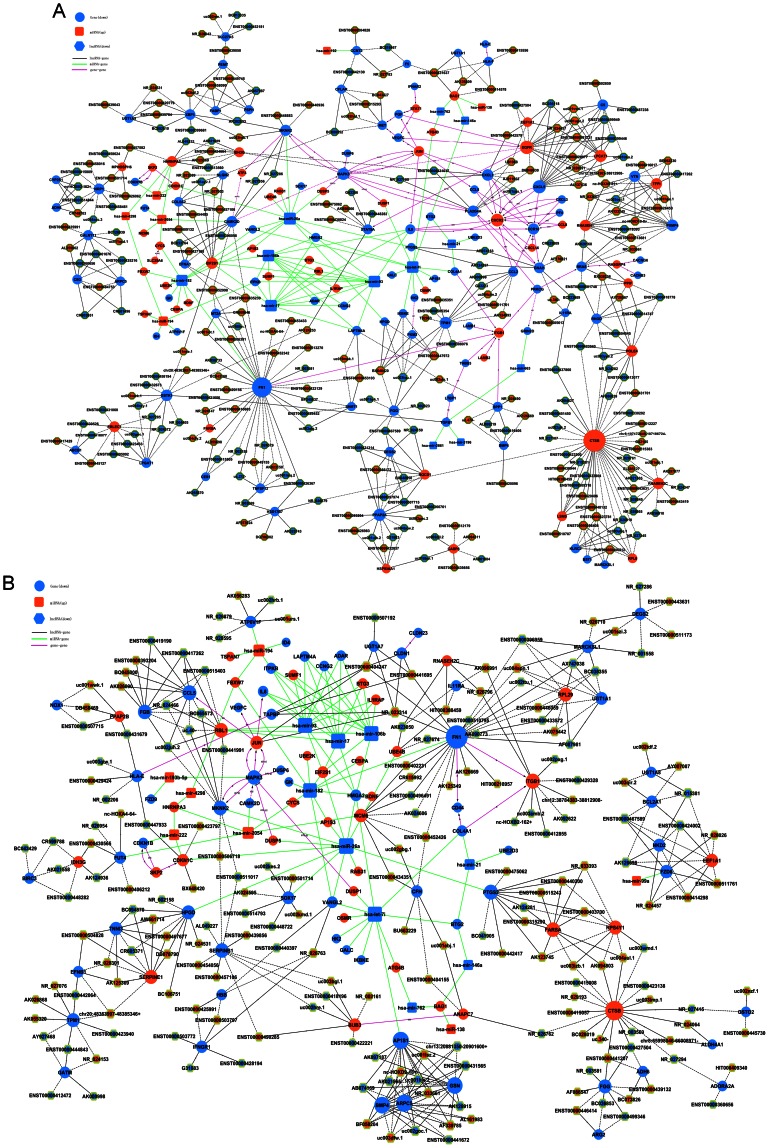
LncRNA-miRNA-mRNA-Network. LncRNA-miRNA-mRNA-Network of A549 (A) and A549/CDDP (B). Blue represents down regulation and orange represents up regulation. Box nodes represent microRNA, circle nodes represent mRNA, and hexagon nodes represent lncRNA. Black edges describe the possible relationship between lncRNA and mRNA, green edges describe the inhibitive effect of microRNA on mRNA, and red edges represent the relationship between mRNA and mRNA.

Pearson correlation coefficients were estimated for each gene and significant correlation pairs were merged with miRNAs to investigate lncRNA, miRNA and mRNA co-expression. Network analysis was undertaken to investigate which gene or genes played a pivotal role in cisplatin resistance (Figure 4). Gene networks were constructed from functional gene associations, and are described in detail in Table S6. mRNAs that interact with both lncRNAs and miRNAs were emphasized with yellow color. In the network diagrams, cycle nodes represent genes, and edges between two nodes represent interactions between genes quantified by degree. Degrees within the network describe the number of single genes that regulate other genes and represent the size of the cycle node. The higher the degree, the more central the gene is within the network. The edges between two nodes were connected by different mechanisms. LncRNA-mRNA was predicted by correlation analysis for losing of effective explanation at present. miRNA-mRNA was predicted by TargetScan which is the most widely used method for high throughput microRNA data targets prediction. mRNA-mRNA was determined based on KEGG for many mRNA relationships had been collected here. LncRNA-lncRNA and miRNA-miRNA were not showed for they are meaningless. LncRNA-miRNA cannot be calculated limited by database and technique.

Clustering coefficients were used to estimate the complexity of interactions among genes neighboring the core gene, with the exception of core gene participation. The lower the clustering coefficient, the more independent of the core genes were in terms of interactions among genes in neighboring cores [35]. The results indicated that many genes like FN1, CTSB, EGFR, and TGFB1; lncRNAs including BX648420, ENST00000366408, and ENST00000404247; and miRNAs such as miR-26a and let-7i potentially played a key role in the network. In the subnetwork, many isolated mRNAs were associated to the Wnt signaling pathway. They were candidates for they may have a significant role in cisplatin resistance regulated by lncRNAs.

The network analysis results suggested that mRNAs can be co-regulated by different lncRNAs together with different miRNAs. For example, BAG1 can be regulated by 3 miRNAs and 3 lncRNAs at the same time. This phenomenon may explain why some mRNAs predicted as potential miRNAs targets did not change with the opposite direction of its corresponding miRNAs.

### Konckdown of AK126698 activated canonical Wnt signaling pathway and induced cisplatin resistance in A549 cell line

**Figure 5:**
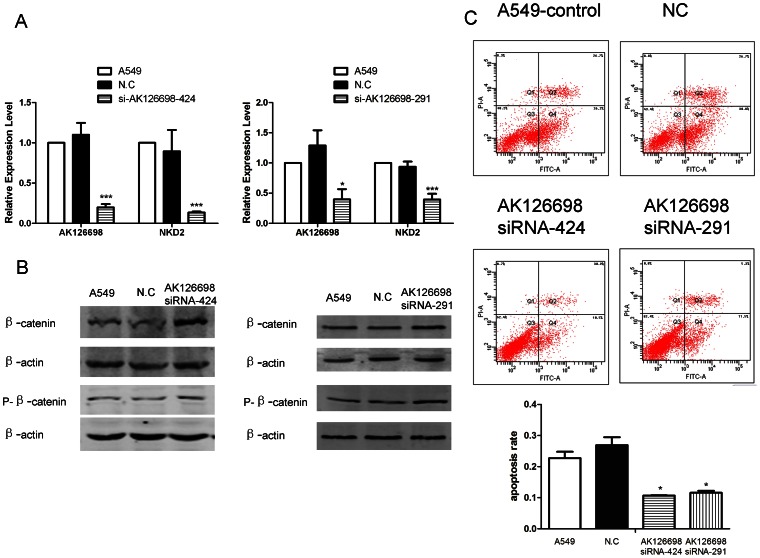
Effects of AK126698 on Wnt pathway and cell apoptosis. Silencing of AK126698 by siRNA in A549 cells and altered NKD2 mRNA expression in AK126698 knockdown A549 cells (A). Western blot analysis of β-catenin and phospho-β-catenin levels in AK126698 knockdown A549 cells (B). Apoptosis evaluated by flow cytometry in AK126698 knockdown A549 cells (C). Values are means±SD and obtained from three independent experiments. *P<0.05, ***P<0.001 compared with A549 and negative control. NC: A549 treated with siRNA negative control; AK126698 siRNA-424, A549 treated with siRNA AK126698-424; AK126698 siRNA-291, A549 treated with siRNA AK126698-291; P-β-catenin, phospho-β-catenin.

The above pathway analyses has showed that Wnt signaling pathway and many isolated members of it listed in relative isolated network were significantly altered in A549/CDDP cell line. These results suggested us that Wnt signaling pathway was involved in NSCLC chemoresistance. Beyond that, the level of AK126698 was closely correlated with many members of the Wnt pathway (as shown in Table S7). Thus, the role of lncRNA AK126698 in regulating the Wnt signaling pathway was explored. Transfection the siRNA AK126698-424 and siRNA AK126698-291 in A549 cells resulted in the decrease of NKD2 mRNA expression which is a negative regulator of Wnt signaling pathway (Figure. 5A). Meanwhile, the key transcription factor in canonical Wnt pathway β-catenin was also increased in protein level after AK126698 knockdown. The protein level of phospho-β-catenin (Ser675), which represents the β-catenin translocation, increased after treatment of siRNA AK126698. These findings suggested that siRNA AK126698 activated canonical Wnt/β-catenin signaling pathway (Figure 5B). At the same time, the effect of knockdown AK126698-424 and siRNA AK126698-291 on the A549 cell apoptosis was observed by using annexin-V/PI assay. As showed in Figure 5C, after exposure to 10 mg/L cisplatin for 24 hours, cells with low AK126698 expression showed stronger resistance to cisplatin-induced apoptosis which implies that downregulation of AK126698 may attribute to the cisplatin resistance in NSCLC maybe thruogh Wnt signal pathway activation.

## Discussion

Cisplatin is an important drug used in lung cancer chemotherapy. However the appearance of chemoresistance impedes its clinical usage. The mechanism for drug resistance involves a series of pathological changes resulting from large-scale gene over-expression and/or under-expression in many cell lines [37], [38], [39]. However, limited understanding of the gene regulation means that our understandings of the mechanisms which underlie cisplatin resistance remain incomplete. In the present study,we evaluated lncRNA and miRNA expression profiles in the cisplatin resistant cell line A549/CDDP. Compared to that of wild type A549 cell line, microarray analysis revealed a set of differentially expressed noncoding RNAs, with 1,380 lncRNAs and 25 miRNAs differentially expressed in A549/CDDP cells. We also identified 1,471 differentially presented protein coding RNAs from the same gene chip.

Pathway analysis, based on the differentially expressed mRNAs from the microarray was developed to shed light on which particular pathways were enriched in genes controlling the distinctive characteristics of A549/CDDP and A549 cells. KEGG annotation showed that many important pathways such as drug metabolism, MAPK signaling, TGF-beta signaling, and Wnt pathway were abundant among significantly enriched mRNAs. And the Western blot results for pathway verification also represent the accuracy of pathway analysis and accordance with previous reports [40], [41], [42]. Most of these pathways have previously been reported to be involved in cisplatin resistance in other cell lines. Wang discovered MAPK-1 is required for cisplatin resistance in human lung cancer cell line H460 [43]. Sun et al had reported toll-like receptor 4 was functionally expressed in human oral squamous cell carcinoma cells and development of resistance to cisplatin [44]. Early in 2002, Tavassoli had proved that cisplatin induced apoptosis was at least partly due to the activation of TGF-beta1 in head and neck cancer cells [45]. And many other pathways such as DNA replication, ABC transporter, Calcium signaling pathway and p53 signaling pathway had also been proved to play roles in the mechanism of cisplatin resistance in various cancer cell lines [46], [47], [48], [49]. Based on the previous reports, we have the reason to believe the results of our pathway analysis.

Currently mostly experimentally confirmed miRNAs targets were based on the conserved 3' UTR pairing. The predicted targets of miRNAs in our list had already been verified in many studies. Trompeter reported RBL1 as a target of both miRNA-17 and miRNA-106b by luciferase experiments [50]. Yu proved miRNA-93 regulates TLE4 on mRNA level and protein level [51]. Such results confirm our predicted targets by TargetScan. So next we attempted to characterize the intersections of differentially expressed mRNAs and the target genes predicted from miRNAs. In the pathway analysis list, 14 pathways were found and some of them had been reported to be regulated by miRNAs in cisplatin resistance. Cheng et al reported miRNA-199a affected cell cycle and increase cisplatin sensitivity [52]. Xu also reported miRNA-M3 suppresses cisplatin-induced apoptosis by targeting TGF-beta signal pathway [53]. This analysis indicated that the Wnt signaling pathway, Toll-like receptor signaling pathway and NOD-like receptor signaling pathway (among others) may be regulated by miRNAs involved in cisplatin resistance.

In biological processes, molecular networks can be constructed using results from co-immunoprecipitation experiments [54] or from the algorithmic predictions based on gene function correlation and expression profiles [55]. The flexibility of network models based on algorithmic predictions from high throughput gene expression tests enables snapshots of gene expression regulatory networks and metabolism pathways among different groups to be obtained. The intrinsic gene networks of a phenotype are thought to represent the gene function propriety of the cell. Such processes may also exhibit regulatory functions. Based on the expression information for lncRNAs and mRNAs, we calculated the Pearson correlation coefficients and chose significant correlation pairs to construct a network. From these results, we identified possible relationships between the noncoding RNAs and mRNAs suggesting that noncoding RNAs maybe have a regulatory impact on the specific mRNAs or vice versa. mRNA plays the direct role in cisplatin resistance, while miRNA and lncRNA perform their functions by epigenetically regulate mRNA. The first purpose of network structure analysis is to locate core regulatory factors (genes). In one network, core regulatory factors connect most adjacent genes and have the biggest degrees. While considering different networks, core regulatory factors were determined by the degree differences between two class samples [56]. They always own the biggest degree differences. Nowadays, only a few studies had confirmed some specific function of lncRNA on regulating mRNAs. Though Rapicavoli reported long noncoding RNA Six3OS acts in trans modulating Six3 activity [57]. The mechanism and function of ncRNAs especially lncRNAs are still unclear. So this network could give us more accurate direction for further investigation. Another main role of the co-expression network is to predict the possible relationship of mRNAs and ncRNAs. Such as mRNA-miRNA target regulation, after negative correlation analysis, many miRNA target genes were screened out for they played the same variational orientation with microRNAs. However, the eliminated relationship of mRNA-miRNA target is affected by many factors possibly including other miRNAs or lncRNAs impact. For instance, mRNA BAG1 and miR-138 were both proved to enhance cisplatin resistance [58], [59], and they had regulation relationship theoretically by TargetScan. But BAG1 changed with the same direction of miRNA-138, which may be influenced by miRNA-762, miRNA-146a or AK125809 that had not been reported to participate in cisplatin resistance. This is another significance of our network.

Investigating genes involved in gene co-expression network, 176 mRNAs, 414 lncRNAs and 20 miRNAs in common were found that may affect cisplatin resistance in NSCLC cell line A549. Among the mRNAs, FN1 and CTSB were identified as the most significant dysregulated genes with largest degrees which may be related to the higher incidence of cisplatin resistance in NSCLC. FN1 previously was thought as playing a role in the cell adhesion and participating in cell metastasis [60]. Recently it was found to be a potential marker of radiation sensitivity in head and neck cancer [61], and also differently expressed in ovarian cancer platinum resistance [62]. Meanwhile it correlated with both lncRNAs and miRNAs which may affect its expression level, FN1 might have an important function in NSCLC cisplatin resistance and regulated by ncRNAs. CTSB was found differently expressed in cisplatin-induced hepatotoxicity recently but its role in cisplatin resistance still needs investigated. Among lncRNA, for almost all of them were lack of function annotation, lncRNAs that have the highest degrees such as BX648420, ENST00000366408, and ENST00000404247 or correlated with the high degree mRNAs such as AK126698 and BC036118 worth further investigation. Among miRNAs, miR-26a and let-7i were identified with highest degrees. MiR-26a was found regulate cell growth and metastasis in many cell lines and favorable on tamoxifen but still lack of its information in cisplatin [63], [64], [65]. Let-7i was found reduced in chemotherapy-resistant ovarian cancer patients which correspond with our results and might be a biomarker to predict chemotherapy response [66].

It is known that coexpression modules may correspond to specific biological pathways. We, therefore, focused on coexpression modules with a high rate of protein-coding RNAs in the coexpression network. Together with the significant pathways identified above, the Wnt signaling pathway appeared in both the mRNA and miRNA significant pathways. It had a relatively independent role in the coexpression network and, for this reason, we propose that the Wnt pathway may play an important role in the process of cisplatin resistance in NSCLC.

The Wnt/β-catenin pathway is one of the most important signal transduction pathways involved in cell growth, differentiation, embryogenesis and oncogenes [67]. It has been reported that overexpression of β-catenin not only plays a role in the NSCLC tumorigenesis but also increases chemoresistance [68]. NKD2 inhibits β-catenin by binding to Dvl protein. Dvl protein is involved in phosphorylation-dependent recruitment of Axin to LRP5 (or LRP6) co-receptors [69]. The sequestration of Axin to the receptor complex results in disassembly of the β-catenin degradation complex and thereby promotes the stabilization and accumulation of cytosolic β-catenin [17]. AK126698 is a 3,826bp in length noncoding RNA exists in cerebellum and defined as human cDNA FLJ44744 fis. It is found by direct sequencing in 2003 and belongs to the subgroup of lincRNA nearby coding gene [70]. Still there is no any data concerning its function of binding or position to date. In our study, we found a correlation between AK126698 and NKD2 (r = 0.999) based on the genechip results. Decreased expression of AK126698 was detected in A549/CDDP cells by both genechip and realtime RT-PCR analysis, suggesting that AK126698 may play an important role in NSCLC cisplatin resistance.

Based on our gene coexpression network, for AK126698 expression level correlated with many members of the Wnt pathway such as NKD2 and FZD8, we supposed it may regulate the expression level of NKD2. Realtime RT-PCR and western blot analysis after knockdown AK126698 in A549 identified decreased expression of NKD2, increased expression of whole β-catenin and nuclear translocation of β-catenin. While, there is no differences in mRNA expression level of β-catenin (CTNNB1) between A549/CDDP cells and wild type A549 cell line in Genechip analysis. These findings suggest that the underlying mechanism of which AK126698 regulating Wnt pathway is not transcriptional activation but may involve reduced degradation of β-catenin via destruction of the complex [71] or other mechanism. Nowadays, epigenetic change had been found in the formation of cisplatin resistance and play an important role. Many CpG sites of the genes acquire methylation in ovarian tumor and gastric cancer cisplatin resistance [72], [73]. And lncRNAs was proved to have the ability recruiting the chromatin modification complexes to particular genomic loci which may be employed for a specific reprogramming of the epigenome of cells. Thus, in this study, the mechanism that AK126698 affects the NKD2 does not the possibility of hypermethylation. But this is still a mechanism conjecture and need further investigate.

In conclusion, the present study shows, for the first time, that numerous ncRNAs are differently expressed between A549 and A549/CDDP cells, many of which may play an important role in regulating cisplatin resistance through various mechanisms including the TGF-beta, ErbB and Wnt pathways. We also found that lncRNA AK126698 regulated A549 cells cisplatin resistance partly through the canonical Wnt pathway. Therefore, sensitizing AK126698 may be an efficient therapeutic intervention for cisplatin resistant for NSCLC. However, the exact mechanism by which AK126698 regulates the Wnt pathway requires further elucidation. And based on these data, further studies of these genes’ expression and function need to be performed in more samples; moreover, the regulation of identified genes and protein functions also should be studied. These further studies will help to improve the clinical treatment and prediction of NSCLC patients.

## Supporting Information

Table S1 Primers of 16 genes selected from gene co-expression network. The table lists the 16 forward primers and reverse primers used for Realtime RT-PCR. The primer for 18S as were used as control for RNA integrity.
(DOC)

Table S2 Sequence of siRNAs used.
(DOC)

Table S3 TargetScan Prediction Results.
(XLS)

Table S4 Intersection part of the predicted target mRNAs and differentially expressed mRNAs.
(XLS)

Table S5 Differently expressed correlated miRNAs and mRNAs.
(DOC)

Table S6 Gene association characters in the gene networks.
(XLS)

Table S7 mRNA/lncRNAs correlated to AK126698. This table lists, RNAs with a correlation coefficient >0.99999 with AK126698 based on the genechip results. Among these, NKD2 and FZD8, which have high correlation coefficients, belong to the Wnt signaling pathway.
(DOC)
