# Supplementary material for: The Noncoding RNA Expression Profile and the Effect of lncRNA AK126698 on Cisplatin Resistance in Non-Small-Cell Lung Cancer Cell
Source: PLoS One. 2013 May 31;8(5):e65309. doi: 10.1371/journal.pone.0065309 (PMC3669360; doi:10.1371/journal.pone.0065309)
Supplement: Table S1 — Primers of 16 genes selected from gene co-expression network. The table lists the 16 forward primers and reverse primers used for Realtime RT-PCR. The primer for 18S as were used as control for RNA integrity. (DOC) [file pone.0065309.s001.doc]

Table S1 Primers of 16 genes selected from gene co-expression network

| **Gene name** | **Primer** | **5’-3’** |
| --- | --- | --- |
| BMP4 | Sense | GATGTGGGCTGGAATGACTG |
|  | Anti-sense | GGTTGGTTGAGTTGAGGTGG |
| CTSB | Sense | TGGTCAACTATGTCAACAAACGG |
|  | Anti-sense | GGTCCTCGGTAAACATAACTCTC |
| NKD2 | Sense | CGTGGATGCCTCGGTCAA |
|  | Anti-sense | GGCTCCTCTGCCAGTTCA |
| BAG1 | Sense | AGCAGTGAACCAGTTGTC |
|  | Anti-sense | GTGTTTCCATTTCCTTCAGAG |
| TGFB1 | Sense | CCCTCCATTCTCCTCTTCC |
|  | Anti-sense | GTCCTCCTTCTGCTCTCCT |
| EGFR | Sense | TCTGCCGCAAATTCCGAG |
|  | Anti-sense | CACCAATACCTATTCCGTTACAC |
| JUN | Sense | CGCATCGCTGCCTCCAA |
|  | Anti-sense | GCTGTGCCACCTGTTCCCT |
| CUL2 | Sense | AACCAGCAACCTTACTCAG |
|  | Anti-sense | CCTGTCTTCCACTTCATTCTC |
| AK126698 | Sense | CTGGCAAGTTCTCATCCACA |
|  | Anti-sense | ATGTTGGCCAAGTTGGTCTC |
| BC045163 | Sense | GCCATCCGTCTTCATCCT |
|  | Anti-sense | CCCAAGCCTCCTAATGCCT |
| AK123263 | Sense | CAAGCTGTGGCCATTCTGTA |
|  | Anti-sense | GGGCAAAGGCACTAATCAAA |
| CES4 | Sense | ACCGTCCAAGCTTCTCATCA |
|  | Anti-sense | GCCCAGAATTTCATCACCAT |
| RP3-508I15.14 | Sense | AACGTCTTCAGGGACGAGAA |
|  | Anti-sense | ACTCTAAGACCTGCCCAGCA |
| TP53TG1 | Sense | ACGAAGGTACCCAACCCTCT |
|  | Anti-sense | GGTGTAAGTGTTCGCCTGGT |
| AC090952.4 | Sense | AACTTTGCCCACATTTCGG |
|  | Anti-sense | GAACTCACACCACTCACGG |
| NCRNA00210 | Sense | GCATTGTGATGATGTGTCCTG |
|  | Anti-sense | GCAAGGTTGTGGTGAGGATT |
| 18S | Sense | GTAACCCGTTGAACCCCATT |
|  | Anti-sense | CCATCCAATCGGTAGTAGCG |

The table lists the 16 forward primers and reverse primers used for Realtime RT-PCR. The primer for 18S as were used as control for RNA integrity.
