# Supplementary material for: The Noncoding RNA Expression Profile and the Effect of lncRNA AK126698 on Cisplatin Resistance in Non-Small-Cell Lung Cancer Cell
Source: PLoS One. 2013 May 31;8(5):e65309. doi: 10.1371/journal.pone.0065309 (PMC3669360; doi:10.1371/journal.pone.0065309)
Supplement: Table S2 — Sequence of siRNAs used. (DOC) [file pone.0065309.s002.doc]

Table S2 Sequence of siRNAs used

| **siRNA name** | **Sequence** | **5’-3’** |
| --- | --- | --- |
| SiRNA-AK126698-341 | Sense | GGCGUAGUUUACAGUAUGUTT |
|  | Anti-sense | ACAUACUGUAAACUACGCCTT |
| SiRNA-AK126698-424 | Sense | CUGCAACCCAAGGAAAUAATT |
|  | Anti-sense | UUAUUUCCUUGGGUUGCAGTT |
| SiRNA-AK126698-1492 | Sense | CCCUUGACUUGGGAUUAAUTT |
|  | Anti-sense | AUUAAUCCCAAGUCAAGGGTT |
| SiRNA-AK126698-291 | Sense | GGGAUACAUACGUACAUAATT |
|  | Anti-sense | UUAUGUACGUAUGUAUCCCTT |
| Negative control | Sense | UUCUCCGAACGUGUCACGUTT |
|  | Anti-sense | ACGUGACACGUUCGGAGAATT |
