# Supplementary material for: The Noncoding RNA Expression Profile and the Effect of lncRNA AK126698 on Cisplatin Resistance in Non-Small-Cell Lung Cancer Cell
Source: PLoS One. 2013 May 31;8(5):e65309. doi: 10.1371/journal.pone.0065309 (PMC3669360; doi:10.1371/journal.pone.0065309)
Supplement: Table S5 — Differently expressed correlated miRNAs and mRNAs. (DOC) [file pone.0065309.s005.doc]

Table S5: Differently expressed correlated miRNAs and mRNAs

| **miRNA** | **genesymbol** | **miRNA.regulation** | **diffgene.regulation** |
| --- | --- | --- | --- |
| hsa-mir-106b | RBL1 | down | Up |
| hsa-mir-106b | EIF2S1 | down | Up |
| hsa-mir-146a | BAG1 | down | Up |
| hsa-mir-17 | RBL1 | down | Up |
| hsa-mir-17 | EIF2S1 | down | Up |
| hsa-mir-182 | UBE2K | down | Up |
| hsa-mir-182 | EIF2S1 | down | Up |
| hsa-mir-182 | FBXW7 | down | Up |
| hsa-mir-192 | CCNT2 | up | down |
| hsa-mir-193b-5p | MAPK3 | up | down |
| hsa-mir-193b-5p | FZD9 | up | down |
| hsa-mir-193b-5p | MARCKSL1 | up | down |
| hsa-miR-194 | ID4 | up | down |
| hsa-miR-194 | NRP1 | up | down |
| hsa-mir-2054 | FUT4 | up | down |
| hsa-mir-2054 | PSPH | up | down |
| hsa-mir-2054 | CAMK2D | up | down |
| hsa-mir-222 | ZFP36 | up | down |
| hsa-mir-222 | CDKN1B | up | down |
| hsa-miR-26a | CDKN1C | down | up |
| hsa-miR-26a | UBE4B | down | up |
| hsa-miR-26a | EIF2S1 | down | up |
| hsa-mir-574-3p | CUL2 | up | down |
| hsa-mir-762 | BAG1 | down | up |
| hsa-mir-93 | RBL1 | down | up |
| hsa-mir-93 | EIF2S1 | down | up |
| hsa-mir-99a | FZD8 | up | down |
