# Supplementary material for: The Noncoding RNA Expression Profile and the Effect of lncRNA AK126698 on Cisplatin Resistance in Non-Small-Cell Lung Cancer Cell
Source: PLoS One. 2013 May 31;8(5):e65309. doi: 10.1371/journal.pone.0065309 (PMC3669360; doi:10.1371/journal.pone.0065309)
Supplement: Table S7 — mRNA/lncRNAs correlated to AK126698. This table lists, RNAs with a correlation coefficient >0.99999 with AK126698 based on the genechip results. Among these, NKD2 and FZD8, which have high correlation coefficients, belong to the Wnt signaling pathway. (DOC) [file pone.0065309.s007.doc]

Table S7. mRNA/lncRNAs correlated to AK126698

| **lncRNA** | **Gene/lncRNA** | **correlation coefficient** |
| --- | --- | --- |
| AK126698 | NKD2 | 0.999999953 |
|  | EEF1A1 | -0.99999957 |
|  | FZD8 | 0.999998248 |
|  | ENST00000424002 | -0.999998017 |
|  | ENST00000467589 | 0.999997138 |
|  | ENST00000414298 | -0.999993851 |
|  | NR_015381 | 0.999993824 |
|  | NR_024457 | 0.999993787 |
|  | BCL2A1 | -0.99999375 |
|  | ENST00000511761 | 0.999991011 |

This table lists, RNAs with a correlation coefficient >0.99999 with AK126698 based on the genechip results. Among these, NKD2 and FZD8, which have high correlation coefficients, belong to the Wnt signaling pathway.
